# Ultra-Narrow Band Mid-Infrared Perfect Absorber Based on Hybrid Dielectric Metasurface

**DOI:** 10.3390/nano9101350

**Published:** 2019-09-20

**Authors:** Sai Chen, Zhao Chen, Junku Liu, Jierong Cheng, Yi Zhou, Lin Xiao, Kai Chen

**Affiliations:** 1 Nanophotonics and Optoelectronics Research Center, Qian Xuesen Laboratory of Space Technology, China Academy of Space Technology, Beijing 100094, China; chensai@qxslab.cn (S.C.); chenzhao@qxslab.cn (Z.C.); liujunku@qxslab.cn (J.L.); 2 Institute of Photonics Technology, Jinan University, Guangzhou 511443, China; 3 Institute of Modern Optics, Nankai University, Tianjin 300350, China; chengjr@nankai.edu.cn; 4 Key Laboratory of infrared imaging materials and detectors, Shanghai Institute of Technical Physics, Chinese Academy of Sciences, Shanghai 200083, China; zhouyi@mail.sitp.ac.cn

**Keywords:** dielectric metasurface, perfect absorber, plasmonics, mid-infrared, sensing

## Abstract

Mid-infrared perfect absorbers (PAs) based on metamaterials have many applications in material analysis and spectral detection thanks to the associated strong light–matter interaction. Most of the PAs are built as ‘metal nanostructure’-insulator-metals (MIM). In this paper, we propose an ultra-narrow band absorber based on dielectric metasurface with a metal film substrate. The absorptance comes from the plasmonic absorption in the metal film, where the absorption is enhanced (while the band of that is compressed) by the super cavity effect of the dielectric metasurface. Based on our numerical calculation, the full-width at half-maximum (FWHM) can reach 67 nm at 8 μm (8‰), which is more than two orders of magnitude smaller than the resonance wavelength and much narrower than the theoretical FWHMs of MIM absorbers. Moreover, we studied their application in infrared thermal imaging, which also has more benefits than MIM absorbers. This kind of hybrid dielectric metasurface provides a new route to achieve ultra-narrow band perfect absorbers in the mid-infrared regime and can be broadly applied in detectors, thermal emitters and bio-spectroscopy.

## 1. Introduction

Infrared (IR) spectroscopy can accurately detect chemical and biological species by characterizing the absorption bands of the chemical bonds they consist of, which are traditionally called the “fingerprints” of the molecules. It has been employed in many research fields, such as pharmaceuticals, industrial inspections, and safety security [1,2,3,4]. In recent years, many perfect absorbers have been proposed for sensing and detection in the infrared and microwave ranges [5,6,7,8,9]. Since the bulk plasmon frequency of metal is far away from these ranges, the surface plasmon resonances of the metallic nanostructure need to be enhanced by a ‘reflective mirror’ to form a sandwich structure as a ‘metal nanostructure–insulator spacer–metal film’ (MIM). With the excitation of surface plasmons, the metallic nanostructures can boost the near field of the electromagnetic waves at specific wavelengths around their surface, which can significantly enhance the interaction between the incident light and the nanostructures or any bound materials on their surface. As the coating or the environment near the structure changes, their spectral response will vary accordingly. Therefore, they have been widely employed for ultrasensitive detection of proteins, other functional molecules, or their structural changes [10,11,12,13,14,15,16] via the so-called “surface-enhanced infrared absorption spectroscopy (SEIRA)” technique. Meanwhile, as they are complementary metal-oxide-semiconductor (CMOS) compatible, they also have great potential to be integrated in micro-electromechanical system (MEMS) devices [17]. Qian et al. applied this in a micromechanical cantilever and proposed zero-power infrared digitizers [18]. However, the performance achieved was still far from the desirable demands in spectroscopy and material research because of the low Q-factors (quality factors) of the plasmon resonances imposed by the resistive loss in the metal [14]. Nanoresonators based on high-index dielectric materials exhibit low intrinsic loss and most of them are also CMOS compatible. They thus provide a new box of tools at subwavelength scale to manipulate the propagation and localization of electromagnetic waves. Many conventional devices, such as lenses, holograms, and antennas, have been redeveloped [19,20,21,22,23,24,25,26]. Recently, Altug et al. proposed pixelated dielectric metasurfaces for mid-infrared spectroscopy sensing [14,27]. Based on the super-cavity modes, driven by Fano resonance or bound states in the continuum [28,29], the metasurface can enhance, detect, and differentiate the absorption fingerprints of various molecules, which opens a new way to enhance mid-IR spectroscopy sensing for chemical or biological detection. 

In this paper, inspired by the super-cavity mode [29], we developed a new type of perfect absorber based on the hybridization of a dielectric metasurface and a metal film substrate. The absorption comes from the plasmonic metal film, which is enhanced by the dielectric metasurface with high Q-factor super-cavity. Ultra-narrow band absorption is achieved in the mid-infrared regime and the full-width at half-maximum (FWHM) can be surpressed below 1% at 8 μm, beyond the theoretical limit of MIM absorbers [17]. Moreover, due to the plasmonic absorption, its capability for integrating into the thermal infrared-detecting chip [30] was studied. The absorption band could be tuned from 4 μm to 16 μm based on different CMOS-compatible dielectric materials and technology, which could serve as a guideline for future research on hyper-spectrally selective infrared detectors and SEIRA chips.

## 2. Methods

**Figure 1:**
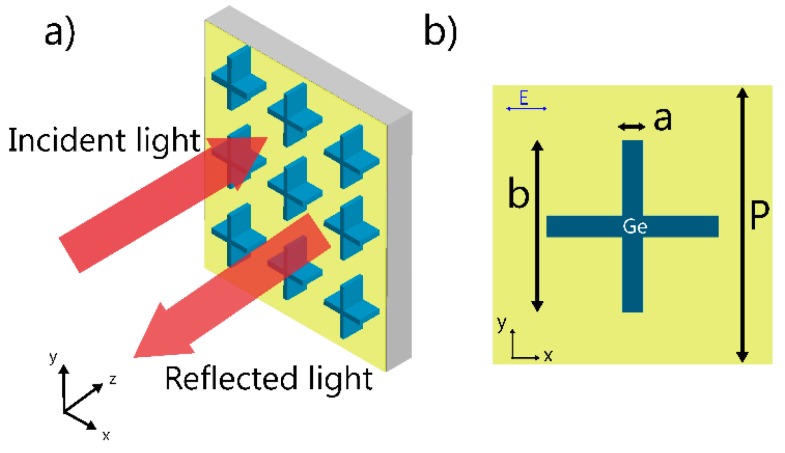
Perspective views of the hybrid metasurface absorber (**a**) and the unit cell (**b**). The height of the resonator is constant at ***h*** = 0.95 μm; the period ***p*** and geometric parameters ***a*** and ***b*** vary for different resonance wavelengths.

Figure 1 shows the geometrical configuration of a representative sample of the studied hybrid dielectric metasurface. Germanium (Ge) cross bars sit on top of a gold (Au) film coating on a silicon substrate. Figure 1b is the unit cell in the finite difference time domain (FDTD) numerical simulation. The incident light is polarized in the *x* direction and propagates along the *z* axis. Periodic boundary conditions are applied in the *x* and *y* directions, while perfectly matched layer (PML) boundary conditions are used in the *z* direction. The electromagnetic wave signal in the time domain becomes long because of the strong narrow-band resonance, and the simulation time is extended to 2500 fs. The height of the cross bar is ***h*** = 0.95 μm. The thickness of the gold film is set to 200 nm with the transmitted mid-infrared light completely blocked (penetration depth δ ≈ 40 nm). Therefore, the reflected light is collected to characterize the optical properties of the metasurface. The dielectric constants of Ge, Au and silicon are obtained from the handbook of Palik [31]. It is noted that the imaginary part of the refractive index of Ge is near zero(<1e^−16^) and the real part is set to 4.01 as there is nearly no dispersion in this range.

In thermal simulation, the heat source distribution is collected from the absorption field and calculated from the metasurface structure. The boundaries of the simulation dimensions are set as closed. The temperature of the ground plane of two isothermal bars is set to 273.15 K, the room temperature. The convection coefficient between air and Ge (Si_3_N_4_ or gold) is set to 10 W/(m^2^·K). The thermal conductivity and specific heat of VOx is 5.75 W/(m·K) and 100 J/(kg·K); the thermal conductivity and specific heat of Si_3_N_4_ is 5.75 W/(m·K) and 100 J/(kg·K); the thermal conductivity and specific heat of Ge is 60 W/(m·K) and 360 J/(kg·K); the thermal conductivity and specific heat of Au is 397 W/(m·K) and 385 J/(kg·K).

## 3. Results and Discussions

### 3.1. Mode Analysis

**Figure 2:**
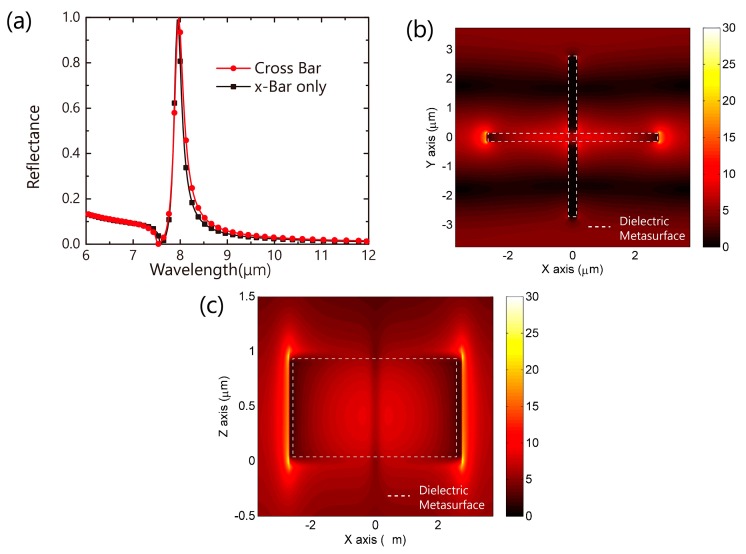
(**a**) The reflectance spectra of the dielectric metasurfaces (cross bar and *x*-bar only) without metal film., A Fano resonance is observed with the full-width at half-maximum (FWHM) 220 nm. The E-field distribution of the metasurface in *x*-*y* plane (**b**) and *x*-*z* plane (**c**) at 7.98 μm. There is a strong E-field localization at the ends of the horizontal bar.

At first step, we studied the pure dielectric metasurface (without the metal film). As shown in Figure 2a, the reflection spectrum of the pure dielectric metasurface exhibits a high Q-factor peak with the period ***p*** = 7.5 μm, geometric parameters ***a*** = 5.5 μm and ***b*** = 0.3 μm. A Fano resonance(R_1_) appears at 7.97 μm. For the dielectric metasurface, it can be treated as a coupling mode between Mie scattering and Fabry–Pérot mode, where the different structure decides the specific transmission mode. At specific height (*z* axis), the metasurface can form a cavity (called resonance) at a specific wavelength band, while tuning the geometric parameters in *x*-*y* plane can compress the band into narrow. At this band, the electromagnetic wave almost reflects due to this cavity. Moreover, some special patterns can be treated as bound states in the continuum, which has an extremely narrow band resonance [28,29]. In our case, based on fruitful geometric freedom, the coupling mode can also be suppressed. The FWHM of the Fano resonance (R_1_) can reach 2.7% of the resonance wavelength (220 nm@7.98 μm). The electric field (E-field) distribution is shown in Figure 2b,c. The electric field is strongly located at the ends of the dielectric arm in the *x*-direction (*x*-Bar) as the incident light is *x*-polarized. We simulated the reflectance spectrum of the *x*-bar-only metasurface. Very similar Fano resonance is observed as shown in Figure 2a. It can be concluded that the cross-bar metasurface can offer polarization independent performance due to its 4-fold symmetry while linear polarization dependence is expected for the *x*-bar-only metasurface. It is noted that this kind of metasurface is incident angle-dependent, and the wavelength of the resonance works in ±5°.

**Figure 3:**
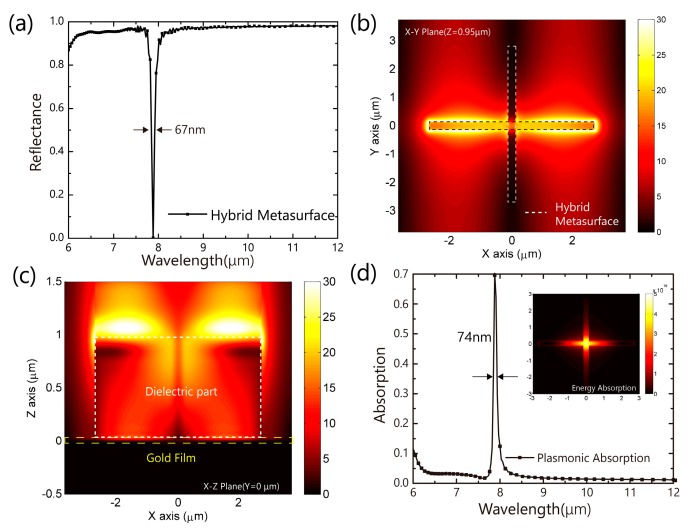
(**a**) The reflectance spectrum of the dielectric metasurface (cross bar with gold film. The FWHM is 67 nm; the E-field distribution of the metasurface at *x*-*y* plane (*z* = 0.95 μm) and *x*-*z* plane (*y* = 0 um) at 7.88 μm is shown in panel (**b**) and (**c**), respectively. There is a strong E-field localization at the edges of the horizontal bar; (**d**) the power absorption spectrum(**d**) of the total structure calculated by the finite difference time domain (FDTD) method. The insets are the absorption distribution at *x*-*y* plane (*z* = −16 nm inside the gold film).

We then studied the hybrid dielectric metasurface (the dielectric metasurface on top of the metal film substrate). Figure 3a shows the reflectance spectrum of the metasurface. The spectrum shows that the light is almost totally reflected except that there is an ultra-narrow band dip at 7.88 μm with FWHM of 67 nm (8.5‰ of 7.88 μm). Compared with the pure dielectric metasurface in Figure 2a, the light incident on the hybrid dielectric metasurface is almost totally reflected, while the reflective Fano resonance become the absorption dip. The spectral position (R_2_) 7.88 μm of the resonance dip is near the Fano resonance wavelength (R_1_) in the pure dielectric metasurface. The small resonance shift comes from the influence of the plasmonic gold film. Based on the dispersion property at mid-IR, the metal film can be treated as a reflectance mirror. In this case, the metal film plays more roles. We simulated the reflectance spectrum of a hybrid dielectric metasurface with a perfect electronic conductor (PEC) film with limited simulation time. There is a strong oscillation at the wavelength R_1,_ which comes from the Fano resonance. Without loss coefficient, the absorption dip is not apparent. Therefore, on the one hand, the metal film acts as a reflectance mirror to enhance the resonance. On the other hand, the metal film provides the loss channel for energy absorbed at the resonance wavelength. Moreover, the loss coefficient of gold is small in the mid-IR range, which also contributes to the observed narrow bandwidth of the absorbing dip. The E-field distribution is shown in Figure 3b,c. Compared with the pure dielectric metasurface, the E-field is confined more tightly and strongly at the edge of the bar. In traditional MIM metamaterial, the absorption is in both metal parts. For this hybrid metasurface, there is nearly no loss in the Ge structure, the energy absorbing (heat generation) does almost come from the gold film. Figure 3d shows that absorption reaches 70% in limited simulation time. 

### 3.2. Tunability

**Figure 4:**
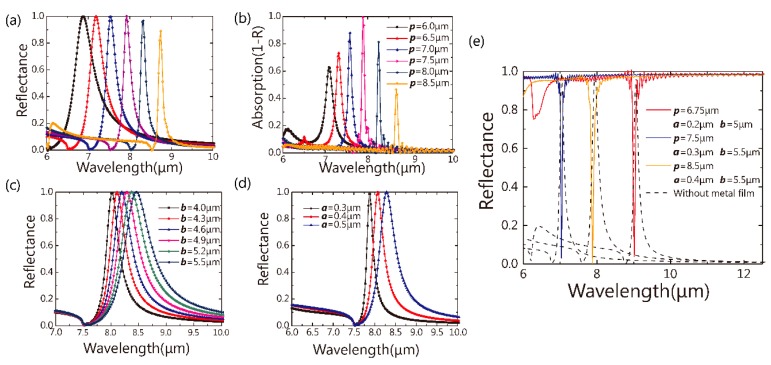
(**a**) The reflectance of the pure dielectric metasurface and (**b**) absorption of the hybrid metasurface (geometric parameters: ***a*** = 0.3 μm, ***b*** = 5.5 μm), while ***p*** varies from 6.0 μm to 8.5 μm; the reflectance spectra (**c**) of the pure dielectric metasurface (***a*** = 0.5 μm, ***p*** = 7.5 μm), while ***b*** varies from 4.0 μm to 5.5 μm; the reflectance spectra(**d**) of the metasurface without gold film (***b*** = 4.6 μm, ***p*** = 7.5 μm), while ***a*** varies from 0.3 μm to 0.5 μm; (**e**) the reflectance of the hybrid metasurface in different parameters (blue, orange and red solid lines) and their corresponding pure dielectric metasurface (black dotted lines).

In dielectric metasurfaces, the geometric parameters determine the spectral positions of the Fano resonances [14]. Therefore, in this part, we studied the effects of the arm’s geometry parameters (***a*** and ***b***) and the period (***p***) on the hybrid metasurface absorber’s resonance. Figure 4a,b show the reflectance spectra of the all-dielectric metasurface and the absorption spectra of the hybrid dielectric metasurface, respectively, with the period ***p*** varying from 6.5 μm to 8.5 μm. For both all- and hybrid-dielectric metasurfaces, the bandwidth of the resonances decrease as the period ***p*** increases. The strength of the resonance of the hybrid metasurface shows larger variations. It can be concluded that the absorption induced by the resonance in a hybrid dielectric metasurface is closely related with the absorption intensity and FWHM of the pure dielectric metasurface. When the Fano resonance in a pure dielectric metasurface is stronger, the absorption and the FWHM of the hybrid metasurface with the same geometries are stronger and narrower respectively. It can be easily understood due to the super-cavity concept coming from the Fabry–Pérot cavity. The influence of the plasmonic film on the FWHM of the absorber’s peak can be treated as a perturbation to the FWHM of the pure dielectric metasurface. Therefore, by studying the pure dielectric metasurface, we could predict the FWHM of the absorber. Due to the strong dispersion of the metal film, the simulation times on the hybrid metasurface are much longer than on the pure dielectric metasurface with the same geometric parameters. If we could only sweep the parameters of the pure dielectric metasurface, the optimization process will be finished quickly saving both time and energy. Figure 4c,d show the reflectance spectra of the pure dielectric metasurface when changing parameters, ***a*** and ***b***. It can be noted that the geometry parameters (***a*** and ***b***) also show a big influence on the FWHM and the intensity of the metasurface.

By using the sweep and optimization algorithm, we could find the proper geometric parameters to achieve ultra-narrow FWHM of the resonance of the pure dielectric metasurface at different wavelengths. Based on these results, we could then select proper parameters for the absorber simulation. In Figure 4e, the FHWM of these absorbers is 100 nm on average, which agrees well with the discussion above. Therefore, by simulating a pure dielectric metasurface, we could design high Q-factor dielectric metasurface perfect absorbers in a more efficient way. It should be noted that a high order of the “cavity” mode exists [32] in Figure 4e, and this could be avoided by changing geometric parameters. 

### 3.3. Potential Applications

**Figure 5:**
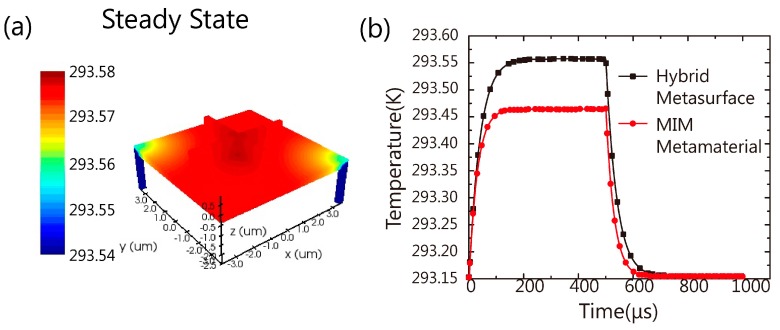
(**a**) The steady-state thermal distribution of the hybrid dielectric metasurface absorber. The power density of the input infrared light is 0.8415 W/cm^2^ (0.473 μw per unit cell). The environmental temperature is 293.15 K, and in order to show the temperature distribution on the metasurface clearly, the lower limit is set to 293.54 K, and the average temperature increase is 0.4 K; (**b**) transient temperature response of the hybrid metasurface absorber and MIM metamaterial bolometer. The input pulse starts from 1 μs to 500 μs. The power density is 0.8415 W/cm^2^_._

For this dielectric metasurface absorber, the required fabrication technique is CMOS compatible. It could be applied in many fields such as emitter [33,34], detector and sensing chips. It could be potentially integrated into infrared thermal detectors. By varying the geometry parameters of the metasurface, we can tune the center wavelength of the absorption dip and the ultranarrow bandwidth of the tip can lead to much better spectral resolution meeting the demands in many fields, such as thermal imaging and material detection. Here, we build a model to demonstrate that this hybrid dielectric metasurface can be integrated into thermal infrared detectors. We used the absorption in Figure 3d as the heat source to simulate thermal radiation and heat transfer. The vanadium oxide (VO_x_) detector is modelled after Du’s work [19,20]. Figure 5a shows the steady-state thermal distribution of the hybrid dielectric metasurface absorber. The temperature of the whole plane above the isolated thermal pillars can reach 293.55K, 0.4 degree higher than room temperature. Meanwhile, the transient state was also studied. As shown in Figure 5b, the temperature reaches the steady state (293.55K) in 151.37 μs. When the input power turns off, the temperature drops to room temperature in 154.15 μs. Moreover, we calculated the transient thermal state of a MIM absorber [30] that exhibits perfect absorption at 9.3 μm with FWHM 3 μm. With the same model of VOx detector structure and input intensity (absorbing energy), the steady temperature of the MIM absorber is 293.45 K. Both absorbers show similar response times to reach the steady state. Compared with the MIM absorber, the hybrid dielectric metasurface reaches 293.45 K faster and shows a higher steady-state temperature. As we discussed above, the absorption in MIM metamaterial happens in the metal structure in both sides of the dielectric layer. As usual, the dielectric layer has low thermal conductivity which means the heat generated in a metal disk cannot transfer down efficiently. In contrast, in our hybrid metasurface, most of the heat is generated in the plasmonic substrate film and can transfer to the VOx more efficiently.

## 4. Conclusions

In summary, we have proposed a new perfect absorber based on a dielectric metasurface with a metal film. The ultra-narrow band absorption comes from the metal’s plasmonic absorption, enhanced and compressed by the super cavity of the dielectric metasurface. The parameters of the dimension are discussed, which shows that its absorption band can be tuned easily by varying the geometry and period of the cross-bar structure. Moreover, the thermal conversion of the hybrid metasurface has been studied. The result shows that it has a great potential to be integrated into infrared thermal detector chips. This kind of hybrid dielectric metasurface provides a new way to produce absorbers at a mid-infrared regime that can be broadly applied in detectors, thermal emitters and bio-spectroscopy.
